# Association of modified dementia risk score with cerebrospinal fluid biomarkers and cognition in adults without dementia

**DOI:** 10.3389/fnagi.2024.1339163

**Published:** 2024-07-16

**Authors:** Qiong-Yao Li, Yan Fu, Xin-Jing Cui, Zuo-Teng Wang, Lan Tan, for the Alzheimer’s Disease Neuroimaging Initiative

**Affiliations:** ^1^Department of Neurology, Qingdao Municipal Hospital, Qingdao University, Qingdao, China; ^2^Department of Outpatient, Qingdao Municipal Hospital, Qingdao, China; ^3^Department of Neurology, Qingdao Hospital, University of Health and Rehabilitation Sciences (Qingdao Municipal Hospital), Qingdao, China

**Keywords:** Alzheimer’s disease, cognition, amyloid-**β**, tau, neuroinflammation, modified dementia risk scores

## Abstract

**Introduction:**

This study aimed to investigate the cognitive profile and prospective cognitive changes in non-demented adults with elevated Modified Dementia Risk Scores (MDRS), while also exploring the potential relationship between these associations and cerebrospinal fluid (CSF) biomarkers of Alzheimer’s disease (AD) pathology and neuroinflammation.

**Methods:**

Within the Chinese Alzheimer’s Biomarker and LifestylE (CABLE) database, 994 participants without dementia were assessed on MDRS, CSF biomarkers and cognition. We examined the associations of the MDRS with CSF biomarkers and cognitive scores using linear regressions. Causal mediation analyses were conducted to analyze the associations among MDRS, brain pathologies, and cognition. The Alzheimer’s Disease Neuroimaging Initiative (ADNI) study was used to validate the mediation effects and to investigate the longitudinal association between MDRS and cognitive decline.

**Results:**

The results revealed that higher MDRS were linked to poorer cognitive performance (Model 1: *P_FDR_* < 0.001; Model 2: *P_FDR_* < 0.001) and increases in CSF levels of phosphorylated tau (P-tau, Model 1: *P_FDR_* < 0.001; Model 2: *P_FDR_* < 0.001), total tau (T-tau, Model 1: *P_FDR_* < 0.001; Model 2: *P_FDR_* < 0.001), P-tau/Aβ42 ratio (Model 1: *P_FDR_* = 0.023; Model 2: *P_FDR_* = 0.028), T-tau/Aβ42 ratio (Model 1: *P_FDR_* < 0.001; Model 2: *P_FDR_* < 0.001) and soluble triggering receptor expressed on myeloid cells 2 (sTrem2, Model 1: *P_FDR_* < 0.001; Model 2: *P_FDR_* < 0.001) in the CABLE study. The impact of MDRS on cognition was partially mediated by neuroinflammation and tau pathology. These mediation effects were replicated in the ADNI study. Baseline MDRS were significantly associated with future cognitive decline, as indicated by lower scores on the Mini-Mental State Examination (MMSE, Model 1: *P_FDR_* = 0.045; Model 2: *P_FDR_* < 0.001), ADNI composite memory score (ADNI-MEM, Model 1: *P_FDR_* = 0.005; Model 2: *P_FDR_* < 0.001), ADNI composite executive function score (ADNI-EF, Model 1: *P_FDR_* = 0.045; Model 2: *P_FDR_* < 0.001), and higher score on the Alzheimer’s Disease Assessment Scale (ADAS13, Model 1: *P_FDR_* = 0.045; Model 2: *P_FDR_* < 0.001).

**Discussion:**

The findings of this study revealed significant associations between MDRS and cognitive decline, suggesting a potential role of tau pathology and neuroinflammation in the link between MDRS and poorer cognitive performance in individuals without dementia. Consequently, the MDRS holds promise as a tool for targeted preventive interventions in individuals at high risk of cognitive impairment.

## 1 Introduction

Dementia has emerged as a pressing global health issue, impacting a substantial number of individuals across the globe (Sommerlad et al., 2019). Alzheimer’s disease (AD) stands as the prevailing etiology of dementia, characterized by progressive cognitive decline(Barnes and Yaffe, 2011). It represents a prominent factor contributing to disability among the elderly population, thereby imposing significant burdens on both patients and society at large (Jia et al., 2020). The ongoing pursuit of efficacious therapeutic approaches aimed at addressing dementia persists (Wang Z. B. et al., 2022; Huang et al., 2023). Hence, there has been a significant emphasis on mitigating the prevalence of individuals affected by dementia. Risk prediction is important for disease prevention, and improved prediction strategies allow for targeted treatment recommendations (Paynter et al., 2010). Over the past two decades, numerous dementia risk prediction models have been devised (Hou et al., 2019). Nevertheless, certain scoring systems encompass intrusive, costly, and inadequately accessible predictors, while others are exclusively applicable to specific populations (Kivipelto et al., 2006; Barnes et al., 2009; Exalto et al., 2014; Amin al Olama et al., 2020). In addition, the 2020 Lancet Commission Report focuses on 12 modifiable risk factors, including low education, smoking, diabetes, depression, physical inactivity, high blood pressure, hearing impairment, obesity, low social contact, excessive alcohol consumption, traumatic brain injury, and air pollution, which they believe that controlling for these 12 factors throughout a human’s lifetime may delay or prevent dementia by 40% (Livingston et al., 2020). In that context, modified dementia risk scores (MDRS) were developed in population data from the UK Biobank, regarded as a potent tool to rapidly identify people at high risk of dementia (Wang Z. T. et al., 2022). MDRS includes easily available risk factors such as age, education, sex, physical activity, current smoking status, glycemic status, and depressive symptoms; and one additionally includes apolipoprotein E (*APOE*) *ε4* carrier status. Currently, cerebrospinal fluid (CSF) and neuroimaging measures of principal pathology of AD are the primary biomarkers supporting the clinical diagnosis of AD (Lantero-Rodriguez et al., 2021). However, these tests have limitations, either requiring invasive lumbar puncture or being too expensive to be widely adopted in non-specialized healthcare settings (Lim et al., 2019). Therefore, researchers have progressively focused on the development of minimally invasive blood biomarkers for screening at the preclinical stage, but there are still problems with the accuracy and stability of current blood biomarkers in diagnosing and predicting AD (Vacinova et al., 2021). Although these biomarkers play a key role in AD diagnosis, they have a limited role in prevention. In contrast, MDRS has the advantage of rapidly identifying people at high risk of dementia and facilitating multiple prevention and intervention for patients against these modifiable factors.

A wealth of evidence indicates that AD-related pathophysiological processes initiate in the brain over a decade prior to the clinical diagnosis of AD, accurately predicting the future progression to AD dementia in non-demented individuals (Barnes and Yaffe, 2011; Sperling et al., 2011; Janelidze et al., 2020). CSF biomarkers can detect these pathologies early and accurately, and their role in AD diagnosis has yielded significant results (Cicognola et al., 2016; Hu et al., 2021). To understand the potential mechanisms underlying the association between the MDRS and dementia, and to further validate the reliability of its predictive power, it is necessary to perform correlation analyses between this risk score and pathological markers. Specifically, decreased levels of CSF amyloid-β (Aβ) 42 and elevated levels of CSF P-tau, CSF T-tau, and CSF soluble triggering receptor expressed on myeloid cells 2 (sTREM2) indicate the presence of Aβ deposition in plaques, neurofibrillary tangles (NFTs), neuronal damage, and neuroinflammation, respectively (Vemuri et al., 2011; Deming et al., 2019). Furthermore, in addition to efforts aimed at identifying risk factors for dementia, there is a growing interest in investigating predictors of cognitive decline, as it is widely acknowledged that dementia has an extended preclinical phase (Kaffashian et al., 2013). The cognitive profiling of individuals identified as high-risk according to the MDRS aids in comprehending the mechanisms that drive the transition from a high-risk state to the clinical presentation of dementia (Kaffashian et al., 2013). Whether and how MDRS also affects cognitive function has not been previously investigated. The correlation between MDRS and cognitive decline may offer valuable insights for the development of strategies to prevent dementia.

The objective of this study was fourfold: (1) to investigate the associations between MDRS and cognition as well as CSF biomarkers in individuals without dementia, (2) to examine whether the impact of MDRS on cognition was mediated by AD pathology in the Chinese Alzheimer’s Biomarker and LifestylE (CABLE) study and the Alzheimer’s Disease Neuroimaging Initiative (ADNI), (3) to explore the connections between MDRS and cognitive decline in the ADNI cohort, and (4) to assess the accuracy of MDRS in distinguishing between AD and cognitively healthy normal controls (NC)/non-dementia subjects in the ADNI cohort.

## 2 Materials and methods

### 2.1 Participants

The study sample consisted of participants from two cohorts. The CABLE study is an ongoing and extensive cohort, which commenced in 2017. The study cohort was centered on investigating the risk factors and biomarkers associated with AD in the Chinese Han population. The participants consisted of Han Chinese individuals aged between 40 and 90 years, who were recruited from Qingdao Municipal Hospital in Shandong Province, China. Exclusion criteria encompassed individuals with a history of central nervous system infection, psychological disorders, severe systemic diseases, or a family history of genetic diseases. A comprehensive evaluation was conducted on all participants, which included structured interviews, questionnaires, biochemical testing, as well as the collection of blood and CSF samples. Demographic data, clinical background, and laboratory results were obtained from electronic medical records. The CABLE study adheres to the principles outlined in the Declaration of Helsinki and has received approval from the Institutional Ethics Committee of Qingdao Municipal Hospital for its research protocol. Prior to participation, written informed consent was obtained from all individuals. To ensure replication and validation, we utilized the Alzheimer’s Disease Neuroimaging Initiative (ADNI) cohort, which was established in 2003 as a collaborative effort between public and private entities. The primary objective of the ADNI study is to establish biomarkers for Alzheimer’s disease (AD) and enhance the comprehension of its pathophysiology. Furthermore, it aims to enhance diagnostic techniques for early detection of AD, improve the design of clinical trials, and investigate the progression rates of mild cognitive impairment and AD. The criteria for inclusion and exclusion in ADNI have been previously outlined (Petersen et al., 2010).

**Figure 1:**
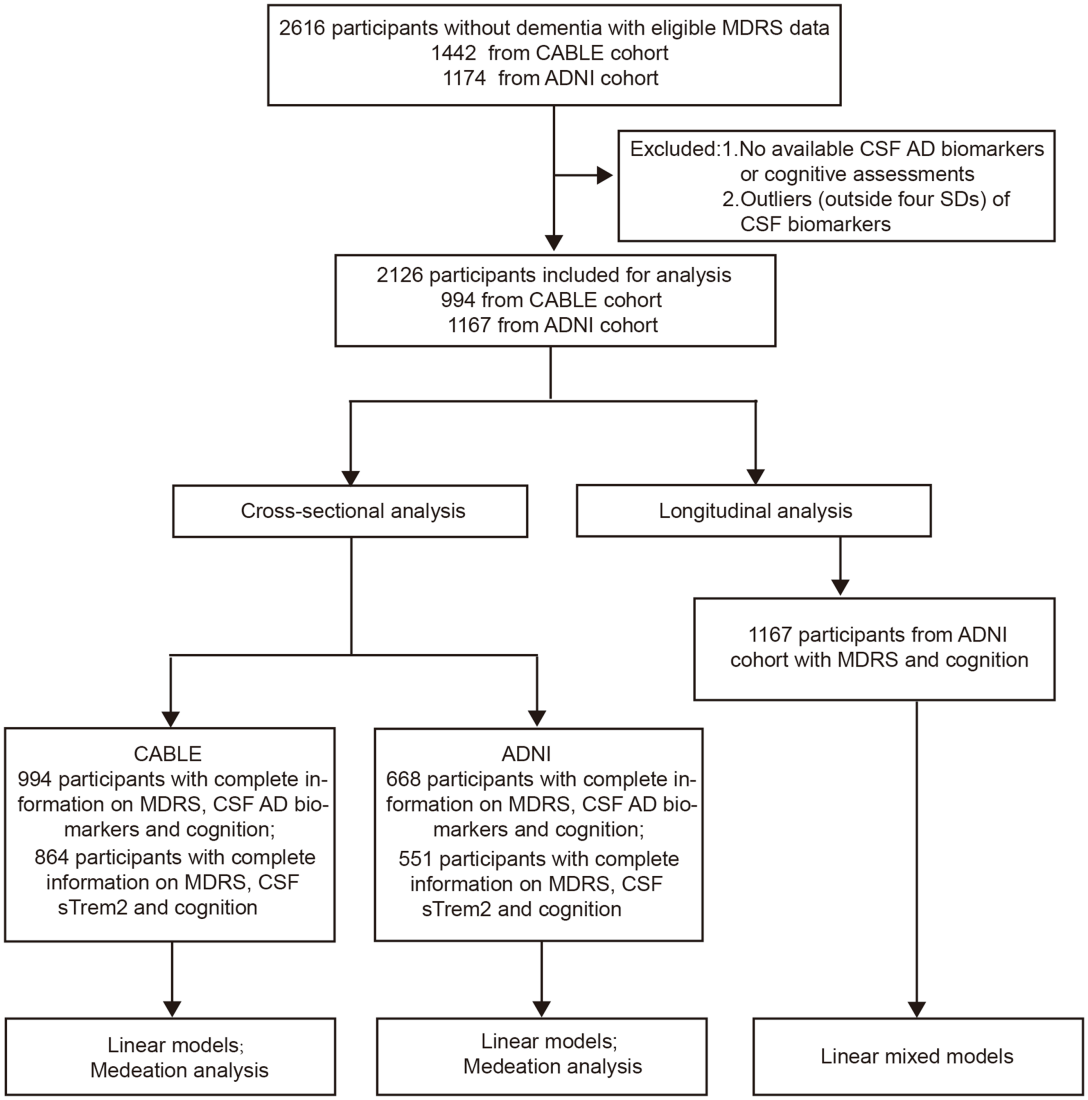
Flow diagram.

In total, the MDRS information of 2,612 participants without dementia were available in these two databases. Among them, participants who underwent CSF biomarkers detection and cognitive assessment were included in the cross-sectional analyses. The longitudinal analyses included participants who completed follow-up cognitive measures for at least 1 year (see Figure 1 for detailed flow diagram of the selection process). Patients with shorter than 1-year follow-up duration were excluded in ADNI study. In addition, 251 patients who were diagnosed with AD at baseline from the ADNI study were included in the analyses to test the accuracy of MDRS in distinguishing between AD and NC/non-dementia subjects.

### 2.2 MDRS calculation

Two risk score models were utilized, with one model incorporating age, education, gender, physical activity, current smoking status, glycemic status, and depressive symptoms, and the other model additionally considering *APOE ε4* carrier status. In the CABLE study, age was classified into five groups (40–48, 49–55, 56–60, 61–64, and > 64 years). Participants were categorized into high (university or above), medium (high school or equivalent), and low (below high school) education level groups. Individuals classified as active engage in leisure time physical activity at least once per week, while those categorized as inactive exercise less frequently than once per week. The smoking status was categorized into current smoking and non-current smoking. The glucose cut-off value of 7.0 mmol/L was selected. The presence of depressive symptoms was defined as a score of >7 points on the Hamilton Depression Rating Scale (HAMDs). *APOE ε4* carrier status was categorized into non-carrier (*APOE ε4*−/−) and carrier (*APOE ε4*+/−, *APOE ε4*+/+). Scores for each factor are summed to provide a total MDRS. And the participants were grouped as high risk (HR) or low risk (LR) for dementia according to the MDRS cutoff of 81 (model 1) or 98 (model 2).

In the ADNI study, age, education, sex, current smoking status were categorized in the same manner as in CABLE. The glucose cut-off value of 126 mg/dL was selected. Depressive symptoms were assessed using Geriatric Depression Scale (GDS), Neuropsychiatric Inventory (NPI), Neuropsychiatric Inventory Questionnaire (NPI-Q). In ADNI 2 and 3, the full NPI was utilized, while in ADNI 1 and ADNI-GO, the shorter NPI-Q was employed. Participants with depression either at the beginning of the study or within the 2 years prior to study entry were excluded, resulting in a limited number of individuals exhibiting significant depressive symptoms at baseline. Depressive symptoms were assessed using the GDS, with scores of 6 or higher indicating the presence of such symptoms. A cut-off of ≥6 was applied for the NPI, and ≥ 2 for the NPI-Q depression sub-scale. Due to the absence of systematically collected data on physical activity, this variable was not included in the risk score.

The score range of MDRS model 1 is 0–154 (age, 0–100 points; education, 0–6 points; sex, 0–10 points; physical activity, 0–3 points; current smoking status, 0–6 points; glycemic status, 0–17 points; depressive symptoms, 0–12 points). The score range of MDRS model 2 is 0–180 (age, 0–100 points; education, 0–6 points; sex, 0–10 points; physical activity, 0–4 points; current smoking status, 0–6 points; glycemic status, 0–16 points; depressive symptoms, 0–12 points; *APOE ε4*, 0–26 points). See Supplementary Table S1 for a detailed description of scoring criteria.

### 2.3 Cognitive measures

In the CABLE study, the assessment of global cognitive function was conducted using a Chinese adaptation of the Mini-Mental State Examination (MMSE). In the ADNI study, cognitive functions were evaluated through the utilization of various scales, such as the MMSE and the Alzheimer’s Disease Assessment Scale (ADAS) for overall cognitive performance, and the examination of specific cognitive domains (executive and memory functions) by reviewing the neuropsychological batteries to identify relevant indicators. In the ADNI cohort, the diagnosis of AD was determined according to the National Institute of Neurological and Communicative Disorders and Stroke–Alzheimer’s Disease and Related Disorders Association criteria for probable AD. More detailed clinical diagnostic criteria are available in the published literature (Petersen et al., 2010).

### 2.4 Measurements of CSF biomarkers

The qualified physician performed lumbar punctures to obtain CSF samples. CSF samples were promptly processed within a two-hour timeframe following their collection. After being centrifuged at a speed of 2000 × g for a duration of 10 min, the sample was divided into smaller portions and stored at a temperature of −80°C to prevent repeated freeze–thaw cycles. The levels of CSF Aβ42, Aβ40, P-tau, and T-tau were determined using ELISA kits (Innotest β-AMYLOID (1–42) [catalog number: 81583]; β-AMYLOID (1–40) [catalog number: 81585]; PHOSPHO-TAU (181p) [catalog number: 81581]; hTAU-Ag [catalog number: 81579]; Fujirebio, Ghent, Belgium), while the level of CSF sTREM2 was measured using the ELISA kit (Human TREM2 SimpleStep ELISA Kit; Abcam, No. Ab224881). All assays were conducted in duplicate by experienced operators who were unaware of the clinical information. The within-batch coefficients of variation (CV) were less than 5%, and the inter-batch CVs were less than 15%.

In the ADNI study, the CSF levels of AD core biomarkers (Aβ42, P-tau, and T-tau) were quantified using the electrochemiluminescence immunoassays Elecsys immunoassays on a cobas 601 instrument. The CSF Aβ42, P-tau, and T-tau measurements were conducted at the ADNI Biomarker Core Laboratory at the University of Pennsylvania using the multiplex xMAP Luminex platform (Luminex Corp, Austin, TX) with Innogenetics (INNO-BIA AlzBio3; Ghent, Belgium; for research use only reagents) immunoassay kit-based reagents. The Alzheimer’s Disease Neuroimaging Initiative provides sTREM2 data from two distinct platforms. One of the sTREM2 datasets is derived from the MSD platform and has been thoroughly described in previous publications (Kleinberger et al., 2014). The adjusted values were utilized and can be accessed in the ADNI database under the variables “MSD_sTREM2corrected.” All CSF biomarker assays were conducted in duplicate and the results were averaged.

### 2.5 *APOE ε4* genotyping assessment

DNA extraction from peripheral blood was performed using the QIAamp^®^ DNA Blood Mini Kit (250), and the DNA samples were stored at −80°C until further analysis. Restriction fragment length polymorphism technology was employed to ascertain the precise loci associated with *APOE ε4* status, namely rs7412 and rs429358. Individuals with at least one *ε4* allele were considered *APOE ε4* carriers.

### 2.6 Statistical analysis

Prior to analysis, outliers of CSF biomarkers, defined as values deviating by 4 standard deviations (SDs) from the mean, were excluded to mitigate the impact of extreme values. In instances where the CSF biomarkers did not conform to a normal distribution, the Box-Cox transformation was utilized to normalize the data. Additionally, Z-score transformation was applied to normalize these data.

To illustrate the basic characteristics of the enlisted participants, we calculated and presented the count and proportion for the categorical variables, along with the average and standard deviation for the continuous variables. Firstly, the disparities in CSF biomarkers between the LR and HR groups were examined using t-test. Secondly, linear regression models were employed to examine the associations between MDRS (as either a continuous or categorical variable) and both cognition and CSF biomarkers. Furthermore, mediation analyses were conducted to assess whether brain pathology mediated the relationship between MDRS and cognition. The indirect effects were estimated, and their significance was determined by utilizing 10,000 bootstrapped iterations. And then, this study employed three mediation models utilizing the structural equation model. The objective of the first mediation model was to examine the potential mediation of P-tau/T-tau in the association between MDRS and cognitive outcome. The second mediation model aimed to investigate whether sTREM2 mediated the relationship between MDRS and P-tau/T-tau. The third mediation model assessed whether sTREM2 and P-tau/T-tau jointly mediated the association between MDRS and cognitive outcome. The relationship between MDRS and cognitive decline was explored using linear mixed-effects models. Lastly, the accuracy of MDRS in distinguishing between AD and NC/non-dementia subjects was evaluated by receiver operating characteristic (ROC) curve and area under the ROC curve (AUC) with 95% confidence intervals (CI). We compared the difference in the AUC of two ROCs using Delong test.

The conventional and significant two-sided *p*-value threshold of 0.05 was utilized. Multiple testing correction was applied using the Benjamini-Hochberg False Discovery Rate (FDR) correction. All statistical techniques and diagram creation were conducted using the R Studio software (version 4.0.5).

## 3 Results

### 3.1 Participant characteristics

Table 1 presents the characteristics of the study population. In CABLE, a total of 994 participants without dementia were included. The average age of participants in this study was 61.99 (± 10.40) years. The mean number of years of education was 9.67 (± 4.24) years. Out of the total participants, 880 had available data on the *APOE ε4* genotype, with 16.80% identified as *APOE ε4* carriers. In ADNI, there were 1,167 non-demented people with MDRS and cognitive information (ADNI 1: *N* = 551; ADNI-GO: *N* = 118; ADNI 2: *N* = 498), and 668 of these participants (ADNI 1: *N* = 232; ADNI-GO: *N* = 83; ADNI 2: *N* = 353) had complete information on CSF biomarkers. These 668 participants all had information on *APOE ε4,* with 48.10% identified as *APOE ε4* carriers. The average age of participants in this study was 73.19 (± 7.09) years. The mean number of years of education was 16.18 (± 2.72) years.

**Table 1:** Basic characteristics of the study population.

	Cable		ADNI (*n* = 668)
Characteristics	All (*n* = 994)	*APOE* genotype available (*n* = 880)	
Age (years)	61.99 (10.40)	61.99 (10.37)	73.19 (7.09)
Gender (male, %)	556 (55.90)	490 (55.70)	388 (58.10)
Education (years)	9.67 (4.24)	9.61 (4.28)	16.18 (2.72)
BMI (kg/m^2^)	25.56 (3.79)	25.58 (3.78)	27.06 (4.69)
Smoke (yes, %)	150 (15.10)	136 (15.50)	97 (14.50)
Physical (inactive, %)	523 (52.60)	466 (53.00)	-
Glucose (mmol/l, mg/dl)	5.80 (1.58)	5.80 (1.59)	100.81 (24.83)
HAMDs	0.54 (1.72)	0.53 (1.71)	–
GDS	–	–	1.41 (1.37)
NPI	–	–	0.44 (1.12)
NPI-Q	–	–	1.19 (0.39)
*APOE ε4* (+, %)	–	148 (16.80)	321 (48.10)
MMSE	27.34 (3.11)	27.29 (3.18)	28.08 (1.78)
ADAS13	–	–	14.42 (7.13)
ADNI-MEM	–	–	0.42 (0.77)
ADNI-EF	–	–	0.35 (0.89)
CSF biomarkers			
Aβ42 (pg/ml)	303.86 (192.02)	297.62 (188.81)	903.05 (366.80)
P-tau (pg/ml)	44.20 (14.03)	43.96 (13.94)	25.83 (12.92)
T-tau (pg/ml)	200.66 (96.37)	200.16 (95.31)	267.10 (116.76)
Aβ42/Aβ40 ratio	0.05 (0.05)	0.05 (0.06)	-
P-tau/Aβ42 ratio	0.22 (0.18)	0.22 (0.18)	0.04 (0.03)
T-tau/Aβ42 ratio	0.97 (0.97)	0.98 (0.88)	0.37 (0.24)
sTrem2 (pg/ml)^*^	18005.12 (7104.00)	18087.31 (7114.95)	3993.62 (2202.00)

APOE genotype was available for 668 participants in ADNI study. BMI, body mass index; HAMDs, Hamilton depression rating scale; GDS, geriatric depression scale; NPI: neuropsychiatric inventory; NPI-Q: neuropsychiatric inventory version Q; APOEꜪ4, apolipoprotein E; MMSE, mini-mental state examination; ADAS: Alzheimer disease assessment scale; ADNI-MEM: ADNI composite memory score; ADNI-EF: ADNI composite executive function score; Aβ42, amyloid β 42; P-tau, phosphorylated tau; T-tau, total tau. ^*^864 participants from CABLE study with CSF sTrem2 information; 770 participants had CSF sTrem2 information among those in the CABLE study for whom APOE genotype information was available; 551 participants from ADNI study with CSF sTrem2 information.

### 3.2 Relationship between MDRS with cognition and CSF biomarkers

**Figure 2:**
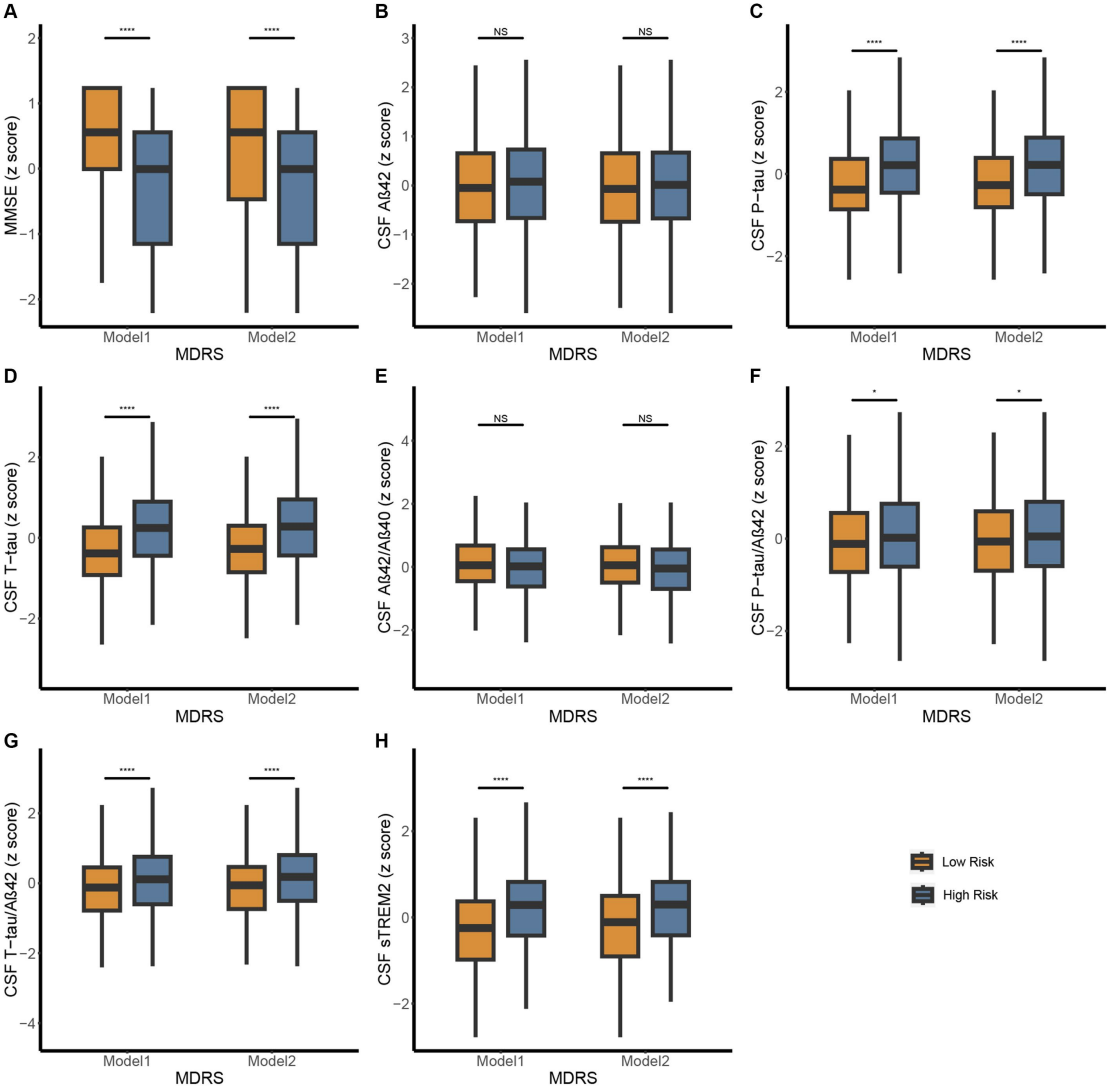
Differences in MMSE and CSF biomarkers between high-risk and low-risk groups defined by MDRS in CABLE study. The patients were stratified into low- and high-risk groups according to the MDRS cutoffs, 81 in model 1 and 98 in model 2. Low risk and high risk were presented using orange and blue, respectively. The figure shows boxplots of each biomarker level in the groups, each showing the median (bar) and interquartile range (whiskers). **(A)** Differences in MMSE between high-risk and low-risk groups defined by MDRS. **(B-H)** Differences in CSF biomarkers between high-risk and low-risk groups defined by MDRS. *p* > 0.05: NS; *p* ≤ 0.05*, *p* ≤ 0.01**, *p* ≤ 0.001***, *p* ≤ 0.0001**** Abbreviations: CSF, cerebrospinal fluid; AD, Alzheimer’s Disease; Aβ, amyloid β; P-tau, phosphorylated tau; T-tau, total tau; sTREM2, soluble triggering receptor expressed on myeloid cells 2; MDRS, modified dementia risk scores; MMSE, Mini-Mental State Examination.

**Figure 3:**
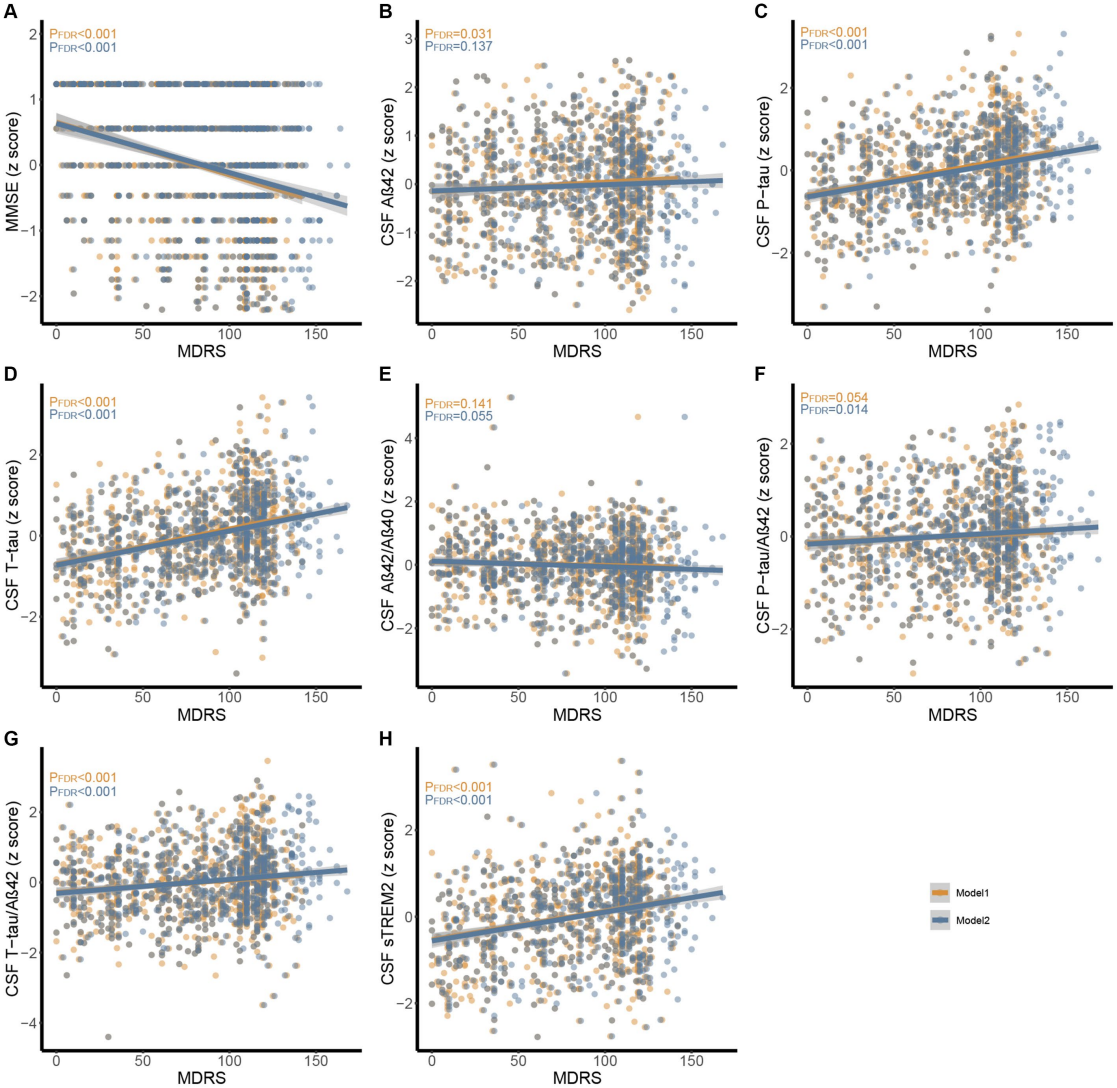
Associations between MDRS with MMSE and CSF biomarkers in CABLE study. Multiple linear regression models were used to examine the associations between the MDRS with MMSE, CSF Aβ42, Aβ40, P-tau, T-tau, Aβ42/Aβ40, P-tau/Aβ42, T-tau/Aβ42, and sTREM2. Model 1 and model 2 were presented using orange and blue, respectively. **(A)** Association between MDRS with MMSE. **(B-H)** Associations between MDRS with CSF biomarkers. Abbreviations: CSF, cerebrospinal fluid; AD, Alzheimer’s Disease; Aβ, amyloid β; P-tau, phosphorylated tau; T-tau, total tau; sTREM2, soluble triggering receptor expressed on myeloid cells 2; MDRS, modified dementia risk scores; MMSE, Mini-Mental State Examination.

In CABLE study, we divided the total population into low dementia risk groups and high dementia risk groups according to the MDRS model 1 cut-off value (LR: MDRS <81, HR: MDRS ≥81) and the MDRS model 2 cut-off value (LR: MDRS <98, HR: MDRS ≥98). The comparison of results across groups was shown in Figure 2. We found that the HR group revealed poorer cognition and higher levels of tau-related biomarkers (P-tau, T-tau, P-tau/Aβ42 ratio, and T-tau/Aβ42 ratio) and sTrem2 compared to the LR group. When MDRS was considered a classification variable, the linear regression showed that HR participants obtained lower MMSE score (Model 1: *β* = −1.499, *P_FDR_* < 0.001; Model 2: *β* = −1.269, *P_FDR_* < 0.001), elevated CSF P-tau (Model 1: *β* = 0.518, *P_FDR_* < 0.001; Model 2: *β* = 0.459, *P_FDR_* < 0.001), T-tau (Model 1: *β* = 0.594, *P_FDR_* < 0.001; Model 2: *β* = 0.570, *P_FDR_* < 0.001), P-tau/Aβ42 ratio (Model 1: *β* = 0.154, *P_FDR_* = 0.023; Model 2: *β* = 0.155, *P_FDR_* = 0.028), T-tau/Aβ42 ratio (Model 1: *β* = 0.278, *P_FDR_* < 0.001; Model 2: *β* = 0.292, *P_FDR_* < 0.001) and sTrem2 (Model 1: *β* = 0.466, *P_FDR_* < 0.001; Model 2: *β* = 0.379, *P_FDR_* < 0.001; Table 2). Due to the missing physical activity data in ADNI study, we did not define the population as high-risk and low-risk based on the MDRS thresholds. When MDRS was considered a continuous variable, the linear regression showed that a higher MDRS was associated with lower MMSE score, elevated CSF P-tau, T-tau, P-tau/Aβ42 ratio, T-tau/Aβ42 ratio and sTrem2 (Figure 3; Supplementary Table S2). Further details were given in Supplementary Table S2. The above meaningful results were replicated in ADNI cohort (Supplementary Figure S1; Supplementary Table S3). It is worth noting that a higher MDRS was associated with lower levels of CSF Aβ42 in model 1 and model 2.

**Table 2:** Associations of modified dementia risk score (as categorical variable) with cognition and CSF biomarkers.

Dependent variables	Modified dementia risk score (Model 1)				Modified dementia risk score (Model 2)			
	*N*	*β*	*p* value	*P_FDR_*	*N*	*β*	*p* value	*P_FDR_*
Cognition								
MMSE	0–80 points (n = 411)	Ref.			0–97 points (n = 452)	Ref.		
	81–154 points (n = 583)	**−1.499**	**<0.001**	**<0.001**	98–180 points (n = 428)	**−1.269**	**<0.001**	**<0.001**
CSF biomarkers								
Aβ42 (pg/ml)	0–80 points (n = 411)	Ref.			0–97 points (n = 452)	Ref.		
	81–154 points (n = 583)	0.094	0.147	0.147	98–180 points (n = 428)	0.058	0.385	0.385
P-tau (pg/ml)	0–80 points (n = 411)	Ref.			0–97 points (n = 452)	Ref.		
	81–154 points (n = 583)	**0.518**	**<0.001**	**<0.001**	98–180 points (n = 428)	**0.459**	**<0.001**	**<0.001**
T-tau (pg/ml)	0–80 points (n = 411)	Ref.			0–97 points (n = 452)	Ref.		
	81–154 points (n = 583)	**0.594**	**<0.001**	**<0.001**	98–180 points (n = 428)	**0.570**	**<0.001**	**<0.001**
Aβ42/Aβ40 ratio	0–80 points (n = 411)	Ref.			0–97 points (n = 452)	Ref.		
	81–154 points (n = 583)	−0.110	0.087	0.100	98–180 points (n = 428)	−0.112	0.101	0.116
P-tau/Aβ42 ratio	0–80 points (n = 411)	Ref.			0–97 points (n = 452)	Ref.		
	81–154 points (n = 583)	**0.154**	**0.017**	**0.023**	98–180 points (n = 428)	**0.155**	**0.021**	**0.028**
T-tau/Aβ42 ratio	0–80 points (n = 411)	Ref.			0–97 points (n = 452)	Ref.		
	81–154 points (n = 583)	**0.278**	**<0.001**	**<0.001**	98–180 points (n = 428)	**0.292**	**<0.001**	**<0.001**
sTrem2 (pg/ml)	0–80 points (n = 361)	Ref.			0–97 points (n = 395)	Ref.		
	81–154 points (n = 503)	**0.466**	**<0.001**	**<0.001**	98–180 points (n = 375)	**0.379**	**<0.001**	**<0.001**

MMSE, Mini-Mental State Examination; Aβ42, amyloid β 42; P-tau, phosphorylated tau; T-tau, total tau; sTREM2, soluble triggering receptor expressed on myeloid cells 2. Bold values indicated statistically significant results.

### 3.3 Causal mediation analyses

**Figure 4:**
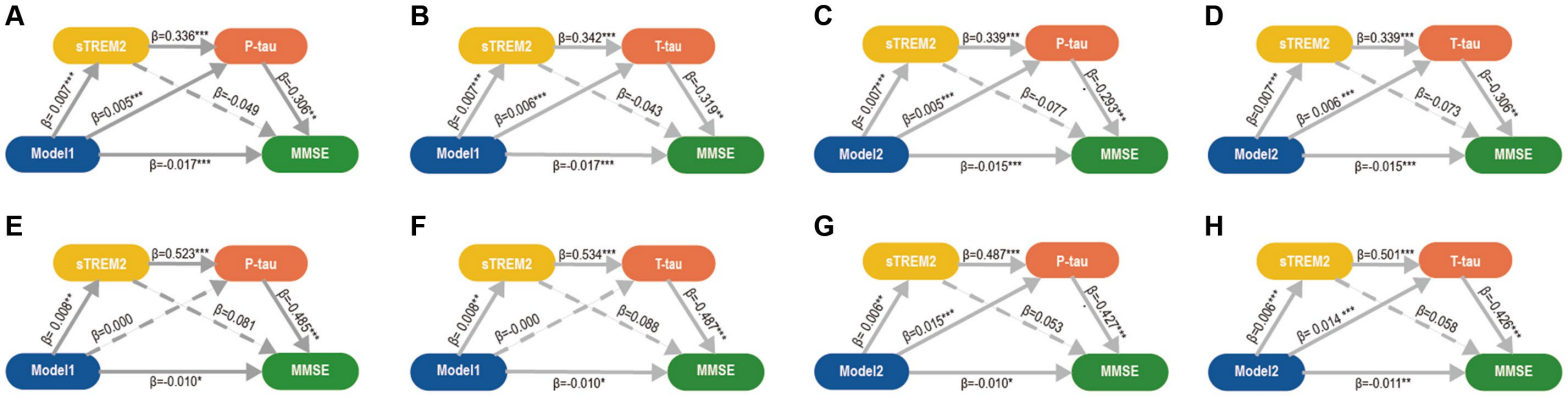
Mediation analyses of CSF biomarkers between MDRS and cognition. Three mediation pathways were performed between MDRS and MMSE: (1) MDRS→sTREM2 → P-tau/T-tau→MMSE; (2) MDRS→sTREM2 → MMSE; and (3) MDRS→P-tau/T-tau→MMSE. **(A-D)**. In CABLE study, the mediation pathways showed that the effect of MDRS via sTREM2 and P-tau/T tau on MMSE was significant. P-tau/T-tau might be a separate significant mediator of this association between MDRS and MMSE, but sTREM2 is not. **(E-H)**. The above findings were validated in ADNI study. *p* ≤ 0.05^*^; *p* ≤ 0.01^**^; *p* ≤ 0.001^***^. MDRS, modified dementia risk scores; AD, Alzheimer’s Disease; P-tau, phosphorylated tau; T-tau, total tau; sTREM2, soluble triggering receptor expressed on myeloid cells 2; MMSE, Mini-Mental State Examination.

Correlations were observed between MDRS, CSF AD core biomarkers, and cognition in two separate cohorts, namely the discovery and validation cohorts (Supplementary Figure S2). We obtain the following analysis results in either the discovery or the validation cohort. The analysis yielded significant results indicating that tau pathology had both direct and indirect effects on cognition. Specifically, the connection between MDRS and MMSE was found to be mediated by tau pathology (Supplementary Figure S3). Additionally, tau pathology was found to mediate the relationship between MDRS and cognitive domains in the ADNI cohort (Supplementary Figure S4). It is noteworthy that Aβ pathology played a role in mediating the relationship between MDRS and cognition in the ADNI cohort (Supplementary Figure S5). Supplementary Figure S6 demonstrated correlations between MDRS, CSF biomarkers, and cognition. Given the intimate association between the sTrem2 and tau pathology, we found that sTrem2 mediate the relationship between MDRS and tau pathology through mediation analysis. We hypothesized that MDRS → sTrem2 → tau pathology → cognition. Therefore, we conducted multiple mediation analysis to verify the hypothesis and obtained the expected results (Figure 4). Similar findings were obtained for global cognition and cognitive domains, including memory and executive functions in ADNI study (Supplementary Figure S7).

### 3.4 Relationship between MDRS with cognitive changes

Subsequently, we examined the association between baseline MDRS and cognitive changes. The interaction of MDRS × time was found to be significantly associated with longitudinal MMSE scores (Model 1: *β* = −0.004, *P_FDR_* = 0.045; Model 2: *β* = −0.012, *P_FDR_* < 0.001) and ADAS13 score for global cognitive scores (Model 1: *β* = 0.010, *P_FDR_* = 0.045; Model 2: *β* = 0.028). Specifically, for memory and executive function, the interaction of MDRS × time was significant in both ADNI-MEM score (Model 1: *β* = −0.001, *P_FDR_* = 0.005; Model 2: *β* = −0.002, *P_FDR_* < 0.001) and ADNI-EF score (Model 1: *β* = −0.001, *P_FDR_* = 0.045; Model 2: *β* = −0.002, *P_FDR_* < 0.001; Table 3).

**Table 3:** Association between MDRS with cognition change in ADNI study.

Cognition	Modified dementia risk score*time (Model 1)				Modified dementia risk score*time (Model 2)			
	*N*	*β*	*p* value	*P_FDR_*	*N*	*β*	*p* value	*P_FDR_*
MMSE	1,167	**−0.004**	**0.045**	**0.045**	1,167	**−0.012**	**<0.001**	**<0.001**
ADAS13	1,167	**0.010**	**0.040**	**0.045**	1,167	**0.028**	**<0.001**	**<0.001**
ADNI-MEM	1,167	**−0.001**	**0.001**	**0.005**	1,167	**−0.002**	**<0.001**	**<0.001**
ADNI-EF	1,167	**−0.001**	**0.022**	**0.045**	1,167	**−0.002**	**<0.001**	**<0.001**

MDRS, Modified Dementia Risk Score; MMSE, mini-mental state examination; ADAS: Alzheimer Disease Assessment Scale; ADNI-MEM: ADNI composite memory score; ADNI-EF: ADNI composite executive function score. Bold values indicated statistically significant results.

### 3.5 Accuracy of MDRS in distinguishing between AD and NC/non-dementia subjects

ROC analysis was performed for 385 NC and 251 AD patients at baseline, and the result showed that MDRS model 2 had an AUC of 0.685 (95% CI = 0.641–0.730), which was superior to model 1 (AUC: 0.474, 95% CI = 0.428–0.520; *p* < 0.001; Supplementary Figure S8A). ROC analysis was performed for 1,174 non-dementia subjects and 251 AD patients at baseline. The ROC analysis yielded similar results (model 2 AUC: 0.633, 95% CI = 0.594-0.672; model 1 AUC: 0.472, 95% CI = 0.433-0.511; *p* < 0.001; Supplementary Figure S8B). In addition, ROC analysis was also used to assess the MDRS’s prediction accuracy. Of the 385 participants who were cognitively normal at baseline, only 5 progressed to AD at follow-up. Of the 1,174 participants who were non-demented at baseline, 314 progressed to AD at follow-up, with a mean length of follow-up of 4.75 years. The results revealed that the predicted AUC values of the model 2 (AUC: 0.674, 95% CI = 0.638-0.709) for predicting AD from non-dementia subjects was still higher than model 1 (AUC: 0.431, 95% CI = 0.394-0.467; *p* < 0.001; Supplementary Figure S8C).

## 4 Discussion

**Figure 5:**
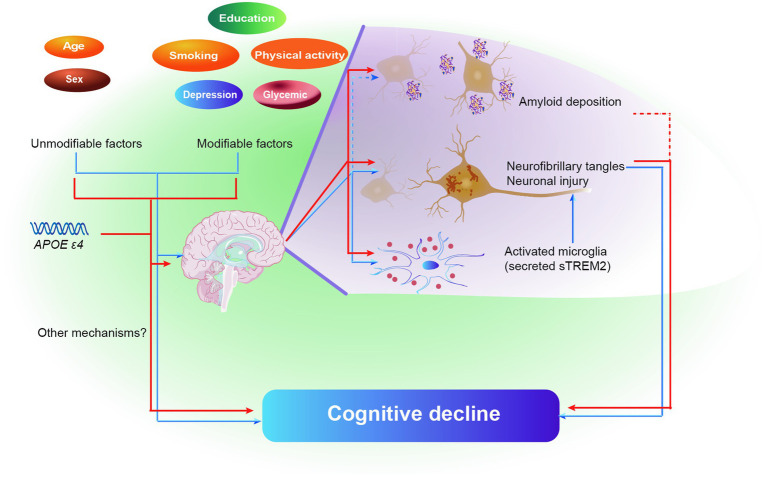
Schematic diagram of the effect of brain pathologies on the association of MDRS and cognitive decline. Blue color represents model 1, and red color represents model 2. Solid lines represent the identical results obtained from two cohorts, and dashed lines represent the results obtained from only ADNI cohort. Abbreviations: MDRS, modified dementia risk scores; AD, Alzheimer’s Disease; Aβ, amyloid β; P-tau, phosphorylated tau; T-tau, total tau; sTREM2, soluble triggering receptor expressed on myeloid cells 2.

The current study discovered that in adults without dementia, MDRS was linked to poorer cognitive performance. The impact of MDRS on cognition was partially influenced by tau pathology and neuroinflammation. Furthermore, a higher MDRS was associated with a more rapid decline in global cognitive score and cognitive domains, including both memory and executive function over time. These findings further solidify the interconnectedness between MDRS, tau pathology, neuroinflammation, and cognition, as depicted in Figure 5. Consequently, these results support the hypothesis that common risk factors, as indicated by MDRS, serve as early markers of dementia risk.

The results of our study align with previous research conducted on the general population, which has shown that the coexistence of multiple risk factors heightens the likelihood of developing dementia. The previous study revealed that these risk factors, namely older age, depression, diabetes, current smoking, and *APOE ε4* carriage, were individually linked to inferior cognitive performance, while protective factors such as higher education levels and vigorous physical activity were associated with better cognitive performance (Lipnicki et al., 2019). Additionally, age, *APOE ε4* carriage, and diabetes were independently associated with cognitive decline (Lipnicki et al., 2019). Research into dementia prevention suggested that a multidomain lifestyle intervention can reduce the risks of cognitive decline and dementia and be beneficial for cognition in elderly people with an elevated risk of dementia (Kivipelto et al., 2018).

Exploring the association between MDRS with neuropathological changes in brain is essential to understand the pathogenesis of poorer cognitive performance in relation to these risk or protective factors. The presence of amyloid accumulation in the neocortex and the aggregation of hyperphosphorylated tau in limbic and cortical association areas are widely recognized as the defining neuropathological features of AD (Khan et al., 2014). While the precise temporal relationship between these core pathologies remains a topic of debate, it is indisputable that tau tangles are strongly associated with neuronal loss and cognitive impairments in both the preclinical and clinical stages of AD (Arriagada et al., 1992; Hardy and Higgins, 1992; Jack et al., 2013; Ossenkoppele et al., 2016; Bierbrauer et al., 2020). It is not surprising that MDRS is significantly associated with tau pathology based primarily on common risk factors for AD. Currently, a few studies focused on the separate components of MDRS, which could provide some support for our results. Aging is widely acknowledged as a significant risk factor for neurodegenerative diseases, such as AD (Mahaman et al., 2022). And under laboratory animal studies, typical AD pathology and neuroinflammation have been demonstrated emergence and progression in an age-dependent manner (Saito et al., 2014). Concurrently, these pathological processes have been shown to induce neuronal atrophy and dysfunction, leading to impaired learning and memory in an age-dependent manner (Dioli et al., 2017; Culig et al., 2022). Prior research has indicated a diminished influence of tau pathology on neuronal function in individuals with higher education who have been diagnosed with AD, thus implying that educational attainment may contribute to the activation of resilience mechanisms (Bierbrauer et al., 2020). Empirical evidence has demonstrated a link between reduced physical activity and heightened deposition of Aβ and neurodegeneration, thereby establishing physical activity as a non-pharmacological intervention with potential cognitive benefits (Brown et al., 2013; Sáez de Asteasu et al., 2019). Smoking has been empirically linked to substantially increased levels of carboxyhemoglobin, impaired functioning of the mitochondrial respiratory chain, and the release of proinflammatory cytokines by glial cells in both the peripheral and central nervous systems. These combined effects ultimately foster considerable cerebral oxidative stress, thereby facilitating abnormal tau phosphorylation within the brain (Durazzo et al., 2014). Certain scholars have posited a correlation between depression and an elevated risk of AD, suggesting that depression may serve as an early indicator of dementia (Singh-Manoux et al., 2017). Depression has been suggested to contribute to tau pathology in previous population studies, and similar conclusions have been reached in autopsy studies (Babulal et al., 2020; Robinson et al., 2021). Furthermore, it is worth noting that during hyperglycemia, there were significant changes in neurotransmitters and degenerative changes in the neurons of the central nervous system (Antony et al., 2010).

The pathology of AD is thought to be related not only to a range of health, genetic, and lifestyle factors but also to inflammation (Javed et al., 2019). Neuroinflammation, characterized by the chronic inflammatory response in the brain, is a prevalent early pathological change in AD (Schain and Kreisl, 2017). Concurrently, the activation of glial cells and neuroinflammation consistently coexist with the presence of tangles and plaques in the cortex (Ingelsson et al., 2004). CSF sTREM2 can be used as a counter for TREM2-triggered microglial cell activity (Ewers et al., 2019). Increased CSF sTREM2 levels were associated with higher CSF P-tau and T-tau concentrations (Suárez-Calvet et al., 2016a,b). As individuals age, microglia become increasingly activated and exhibit a diminished capacity to phagocytose tau, while also generating reactive oxygen species and releasing pro-inflammatory cytokines. These processes further contribute to tau phosphorylation (Busche and Hyman, 2020). In some preclinical studies, animals prone to neuroinflammation showed more severe neurofibrillary tau pathology than animals with less severe neuroinflammation (Stozicka et al., 2010; Jack et al., 2013). In addition, some investigators have found that P-tau pathology progresses very slowly in the absence of microglia (Shi et al., 2019). These findings provided support for the rationality of our observations. From our results, it appeared that these risk factors included in MDRS may promote inflammation and can act in conjunction with inflammation to affect tau pathology in the brain, ultimately leading to cognitive decline.

Our study did not ascertain the impact of MDRS on cognitive functions that were influenced by amyloid pathology. There was no significant association between MDRS with amyloid pathology in the CABLE study, but in the ADNI study, MDRS was associated with lower CSF Aβ42 level. We considered that this result was due to a greater proportion of *APOE ε4* carriers (48.1%) in ADNI study. This association can be attributed to the well-established role of the *APOE ε4* allele in Aβ deposition. The *APOE ε4* allele is widely recognized as the most significant genetic risk factor for sporadic, late-onset AD, significantly increasing the likelihood of developing AD (Castellano et al., 2011). *APOE ε4* has been found to be linked to greater Aβ deposition (Vemuri et al., 2010). On one hand, *APOE ε4* could stimulate Aβ production, and exacerbate amyloid pathology during the initial Aβ seeding stage. On the other hand, *APOE ε4* might affect the clearance pathway of Aβ pathology in the brain, such as enzymatic degradation, cellular uptake, subsequent degradation, clearance through the blood–brain barrier, and so on (C.C. Liu et al., 2013; Yamazaki et al., 2019). In the CABLE study, the proportion of *APOE ε4* carriers was only 16.8%. It is plausible that this phenomenon is specific to the Chinese population. We examined the demographic characteristics of other cohort studies conducted in China and discovered that the proportion of *APOE ε4* carriers is comparatively low within the Chinese population. For example, in the community-sourced Shandong Yanggu Study of Aging and Dementia (SYS-AD) study, the proportion of *APOE ε4* carriers was 15.5% (R. Liu et al., 2022). Similarly, the multicenter study-the China Aging and Neurodegenerative Initiative (CANDI) study reported a proportion of 18.3% (Gao et al., 2023). And in the national cohort study-Chinese Longitudinal Healthy Longevity Survey (CLHLS), the proportion of *APOE ε4* carriers was found to be 17.5% (Jin et al., 2021). Furthermore, when comparing the efficacy of the two models in discriminating and predicting AD, the results also imply an important role for the *APOE ε4* factor. In the results of the ROCs, we found that the MDRS was less efficacious in distinguishing AD from NC/non-dementia subjects. We hypothesized three reasons for this result. Firstly, there was a lack of physical activity data in the ADNI database. Secondly, the duration of participant follow-up differed between the two databases, with a mean follow-up age of 4.75 years in ADNI database and 8.67 years in UKB database. Thirdly, there was a large difference in the sample sizes of the ADNI database and UKB database.

The current study possesses several limitations. Firstly, the data pertaining to MDRS primarily relied on self-reported questionnaires, thereby introducing potential response bias. Secondly, there exists significant variability in the proportions of *APOE ε4* carriers among the CABLE and ADNI cohorts, necessitating further investigations to validate our findings and assess the correlation between MDRS and Aβ pathology. Thirdly, due to the absence of physical activity data in the ADNI database, we were unable to classify the population as high-risk or low-risk based on the MDRS thresholds.

In conclusion, a higher MDRS was found to be linked to poorer cognitive functioning, potentially attributable to its association with tau-related pathologies and neuroinflammation. Given the observed correlation between MDRS and future cognitive score changes in non-demented individuals, it could serve as a predictive indicator for cognitive decline. By managing potentially modifiable risk or protective factors (for example, education, physical activity, current smoking, glycemic, and depressive), it is possible to mitigate or reduce cognitive decline associated with the disease.

## Data availability statement

The CABLE datasets used during the current study are available from the corresponding author on reasonable request. The dataset supporting the conclusions of this article is available in the ADNI site, http://adni.loni.usc.edu/.

## Ethics statement

In the CABLE study, the study involving humans was approved by the Institutional Ethics Committee of Qingdao Municipal Hospital. The study was conducted in accordance with the local legislation and institutional requirements. The participants provided their written informed consent to participate in this study. ADNI was approved by the institutional review boards of all participating institutions. All participants provided written informed consent according to the Declaration of Helsinki before study enrollment.

## Author contributions

Q-YL: Writing – original draft. YF: Methodology, Writing – original draft. X-JC: Methodology, Writing – review & editing. Z-TW: Methodology, Writing – review & editing. LT: Writing – review & editing.
