# Supplementary material for: Association of modified dementia risk score with cerebrospinal fluid biomarkers and cognition in adults without dementia
Source: Front Aging Neurosci. 2024 Jul 16;16:1339163. doi: 10.3389/fnagi.2024.1339163 (PMC11286572; doi:10.3389/fnagi.2024.1339163)
Supplement: Supplementary file 1 [file Data_Sheet_1.docx]

Supplementary Material

**Supplementary Table 1. Modified Dementia Risk Score models used in the study**

**Supplementary Table 2. Association between MDRS with cognition and CSF biomarkers in CABLE study**

**Supplementary Table 3. Association between MDRS with cognition and CSF biomarkers in ADNI study**

**Supplementary Figure 1. Associations between MDRS with cognition and CSF biomarkers in ADNI study**

**Supplementary Figure 2. Correlations between MDRS, CSF AD core biomarkers and cognition in CABLE and ADNI study**

**Supplementary Figure 3. CSF tau-related biomarkers moderated the effects of MDRS on MMSE in CABLE and ADNI study**

**Supplementary Figure 4. CSF tau-related biomarkers moderated the effects of MDRS on ADAS and cognitive domains as cognitive outcomes in ADNI study**

**Supplementary Figure 5. CSF Aβ42 moderated the effects of MDRS on MMSE, ADAS and cognitive domains as cognitive outcomes in ADNI study**

**Supplementary Figure 6. Correlations between MDRS, CSF biomarkers and cognition in CABLE and ADNI study**

**Supplementary Figure 7. CSF sTrem2 and tau-related biomarkers moderated the effects of MDRS on ADAS and cognitive domains as cognitive outcomes in ADNI**

**Supplementary Figure 8.** **Accuracy of MDRS in distinguishing between AD and NC/****non-dementia subjects**

**Supplementary Table 1.** Modified Dementia Risk Score models used in the study

| Risk factors | Modified Dementia Risk Score | |
| --- | --- | --- |
|  | Model1 (Without *APOE* ε4) | Model2 (With *APOE* ε4) |
| Age |  |  |
| 40-48 | 0 | 0 |
| 49-55 | 26 | 26 |
| 56-60 | 55 | 55 |
| 61-64 | 76 | 76 |
| ﹥64 | 100 | 100 |
| Education |  |  |
| High | 0 | 0 |
| Intermediate | 4 | 4 |
| Low | 6 | 6 |
| Sex |  |  |
| Women | 0 | 0 |
| Men | 10 | 10 |
| Physical activity |  |  |
| Active | 0 | 0 |
| Inactive | 3 | 4 |
| Current smoking status |  |  |
| No | 0 | 0 |
| Yes | 6 | 6 |
| Glycemic status |  |  |
| ≤7.0 mmol/L/ ≤126mg/dl | 0 | 0 |
| >7.0 mmol/L/ >126mg/dl | 17 | 16 |
| Depressive symptoms |  |  |
| No | 0 | 0 |
| Yes | 12 | 12 |
| *APOE* ε4 status |  |  |
| Non–ε4 | - | 0 |
| ε4 | - | 26 |
| Total points | 154 | 180 |

**Supplementary Table 2.** Association between MDRS with cognition and CSF biomarkers in CABLE study

|  | Modified Dementia Risk Score (Model 1) | | | |  | Modified Dementia Risk Score (Model 2) | | | | |
| --- | --- | --- | --- | --- | --- | --- | --- | --- | --- | --- |
|  | N | β | *P* value | *P_FDR_* |  | N | β | *P* value | *P_FDR_* |  |
| Cognition |  |  |  |  |  |  |  |  |  |  |
| MMSE | 994 | **-0.021** | **<0.001** | **<0.001** |  | 880 | **-0.020** | **<0.001** | **<0.001** |  |
| CSF biomarkers |  |  |  |  |  |  |  |  |  |  |
| Aβ42 (pg/ml) | 994 | **0.002** | **0.023** | **0.031** |  | 880 | 0.001 | 0.137 | 0.137 |  |
| P-tau (pg/ml) | 994 | **0.007** | **<0.001** | **<0.001** |  | 880 | **0.007** | **<0.001** | **<0.001** |  |
| T-tau (pg/ml) | 994 | **0.009** | **<0.001** | **<0.001** |  | 880 | **0.008** | **<0.001** | **<0.001** |  |
| Aβ42/Aβ40 ratio | 994 | -0.001 | 0.141 | 0.141 |  | 880 | **-0.002** | **0.048** | 0.055 |  |
| P-tau/Aβ42 ratio | 994 | **0.002** | **0.047** | 0.054 |  | 880 | **0.002** | **0.011** | **0.014** |  |
| T-tau/Aβ42 ratio | 994 | **0.004** | **<0.001** | **<0.001** |  | 880 | **0.004** | **<0.001** | **<0.001** |  |
| sTrem2 (pg/ml) | 864 | **0.007** | **<0.001** | **<0.001** |  | 770 | **0.007** | **<0.001** | **<0.001** |  |

Abbreviations: MDRS, Modified Dementia Risk Score; MMSE, mini-mental state examination; Aβ42, amyloid β 42; P-tau, phosphorylated tau; T-tau, total tau; sTREM2, soluble triggering receptor expressed on myeloid cells 2.

**Supplementary Table 3.** Association between MDRS with cognition and CSF biomarkers in ADNI study

|  | Modified Dementia Risk Score (Model 1) | | | |  | Modified Dementia Risk Score (Model 2) | | | |
| --- | --- | --- | --- | --- | --- | --- | --- | --- | --- |
|  | N | β | *P* value | *P_FDR_* |  | N | β | *P* value | *P_FDR_* |
| Cognition |  |  |  |  |  |  |  |  |  |
| MMSE | 668 | **-0.014** | **0.001** | **0.003** |  | 668 | **-0.019** | **<0.001** | **<0.001** |
| ADAS13 | 668 | **0.067** | **<0.001** | **0.001** |  | 668 | **0.097** | **<0.001** | **<0.001** |
| ADNI-MEM | 668 | **-0.008** | **<0.001** | **<0.001** |  | 668 | **-0.011** | **<0.001** | **<0.001** |
| ADNI-EF | 668 | **-0.013** | **<0.001** | **<0.001** |  | 668 | **-0.011** | **<0.001** | **<0.001** |
| CSF biomarkers |  |  |  |  |  |  |  |  |  |
| Aβ42 (pg/ml) | 668 | **-0.006** | **0.012** | **0.014** |  | 668 | **-0.017** | **<0.001** | **<0.001** |
| P-tau (pg/ml) | 668 | **0.006** | **0.026** | **0.028** |  | 668 | **0.018** | **<0.001** | **<0.001** |
| T-tau (pg/ml) | 668 | **0.005** | **0.042** | **0.042** |  | 668 | **0.017** | **<0.001** | **<0.001** |
| P-tau/Aβ42 ratio | 668 | **0.007** | **0.004** | **0.005** |  | 668 | **0.020** | **<0.001** | **<0.001** |
| T-tau/Aβ42 ratio | 668 | **0.007** | **0.004** | **0.005** |  | 668 | **0.020** | **<0.001** | **<0.001** |
| sTrem2 (pg/ml) | 548 | **0.008** | **0.002** | **0.004** |  | 548 | **0.006** | **0.005** | **0.005** |

Abbreviations: MDRS, Modified Dementia Risk Score; MMSE, mini-mental state examination; ADAS: Alzheimer Disease Assessment Scale; ADNI-MEM: ADNI composite memory score; ADNI-EF: ADNI composite executive function score; Aβ42, amyloid β 42; P-tau, phosphorylated tau; T-tau, total tau; sTREM2, soluble triggering receptor expressed on myeloid cells 2.


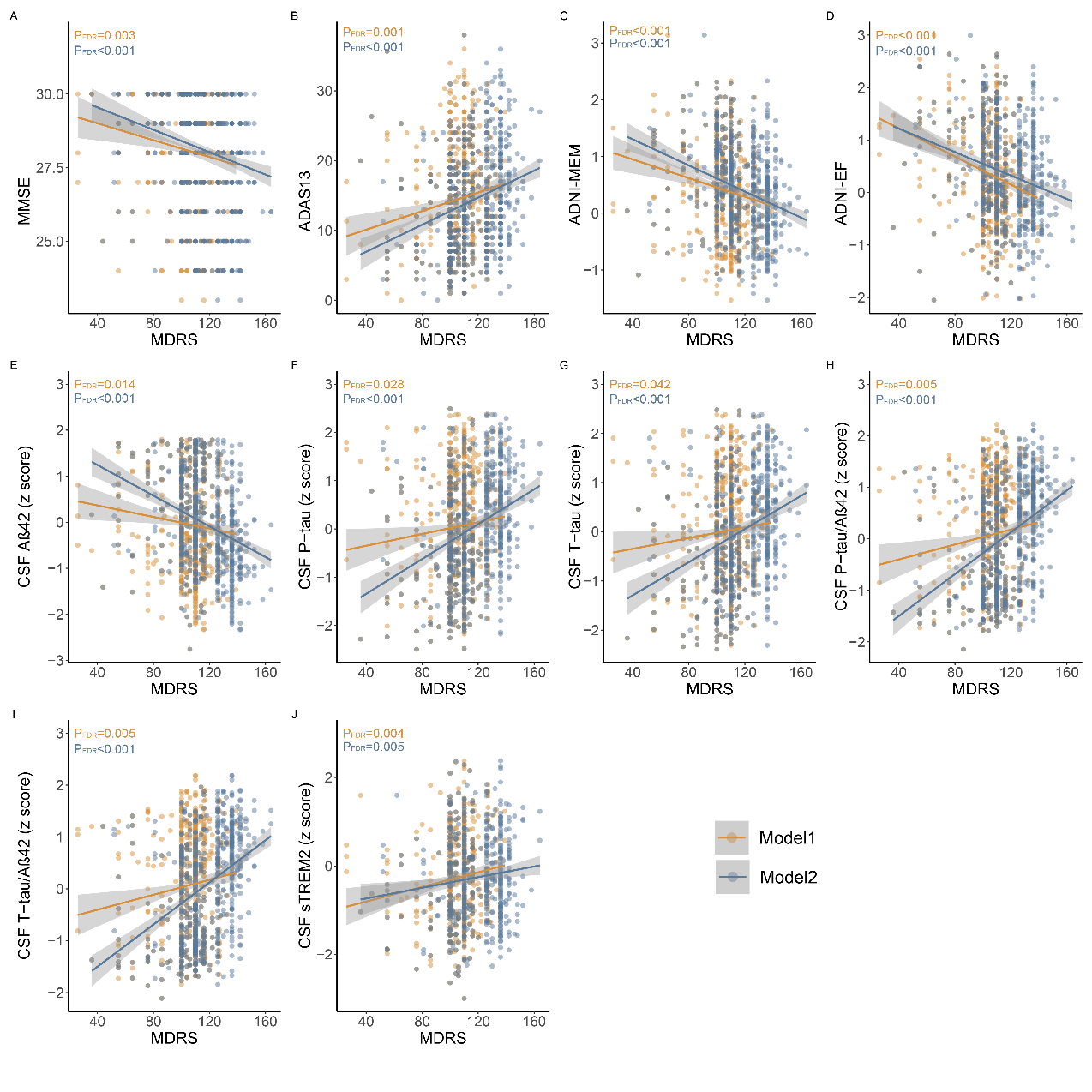

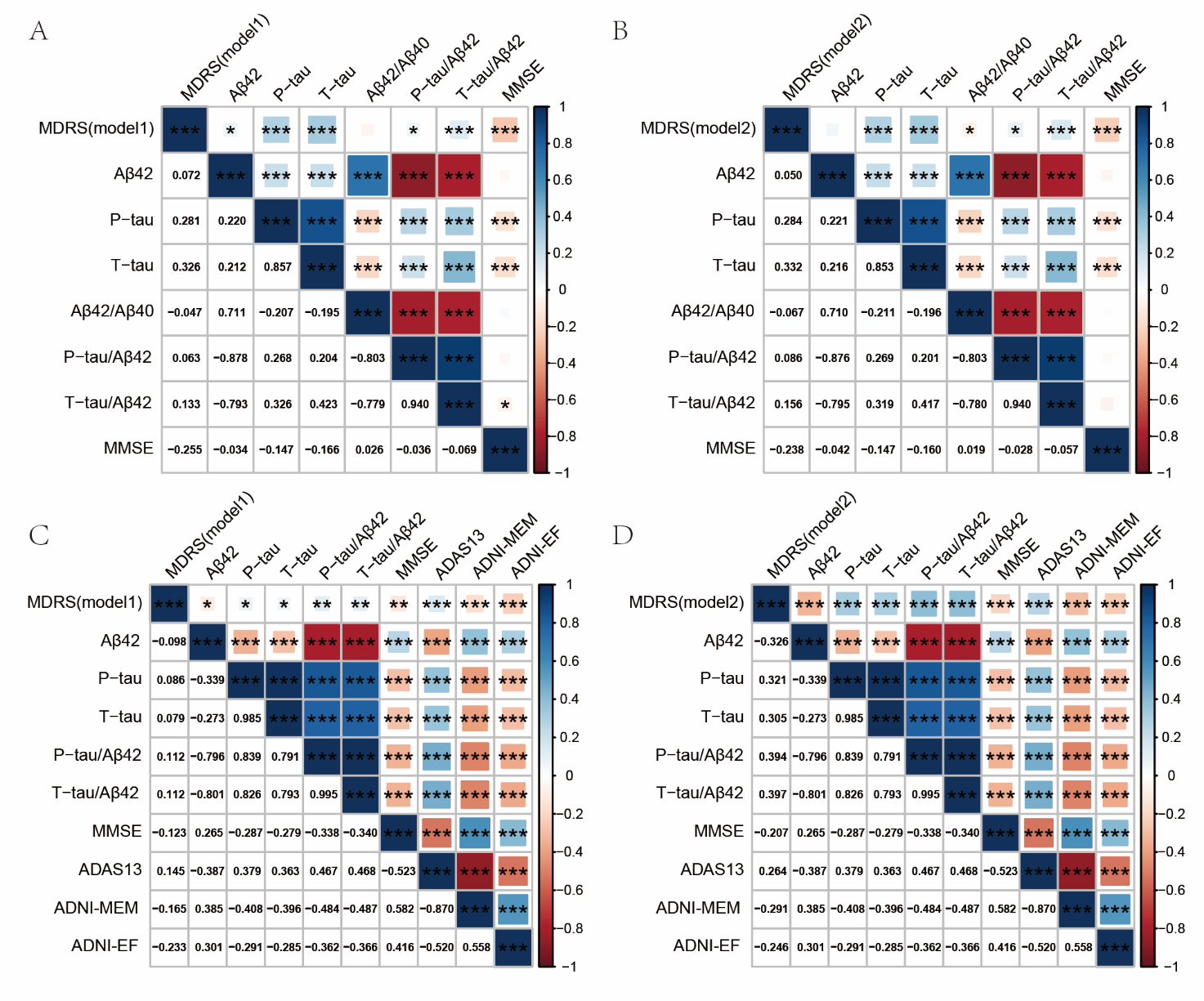


Supplementary Figure 1. Associations between MDRS with cognition and CSF biomarkers in ADNI study

Supplementary Figure 2. Correlations between MDRS, CSF AD core biomarkers and cognition in CABLE and ADNI study


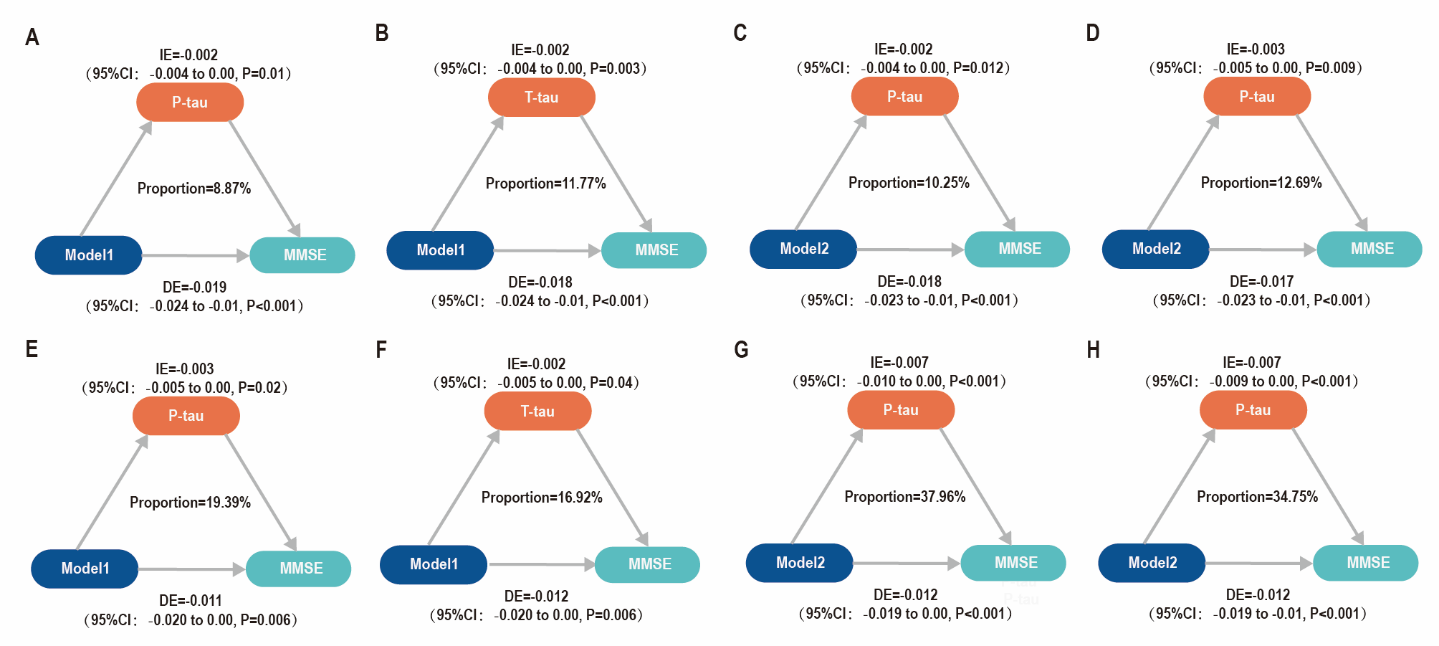


Supplementary Figure 3. CSF tau-related biomarkers moderated the effects of MDRS on MMSE in CABLE and ADNI study


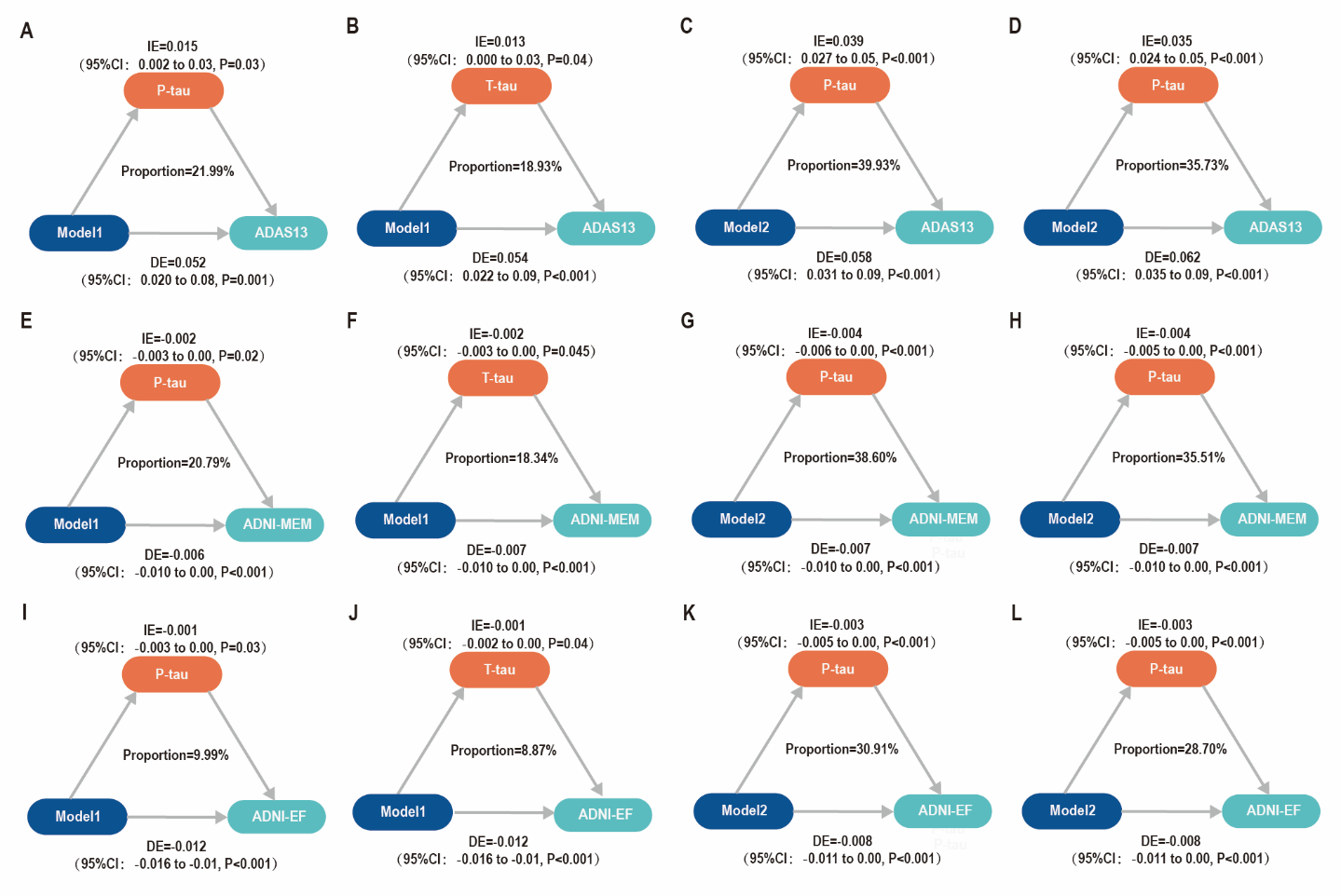


Supplementary Figure 4. CSF tau-related biomarkers moderated the effects of MDRS on ADAS and cognitive domains as cognitive outcomes in ADNI study


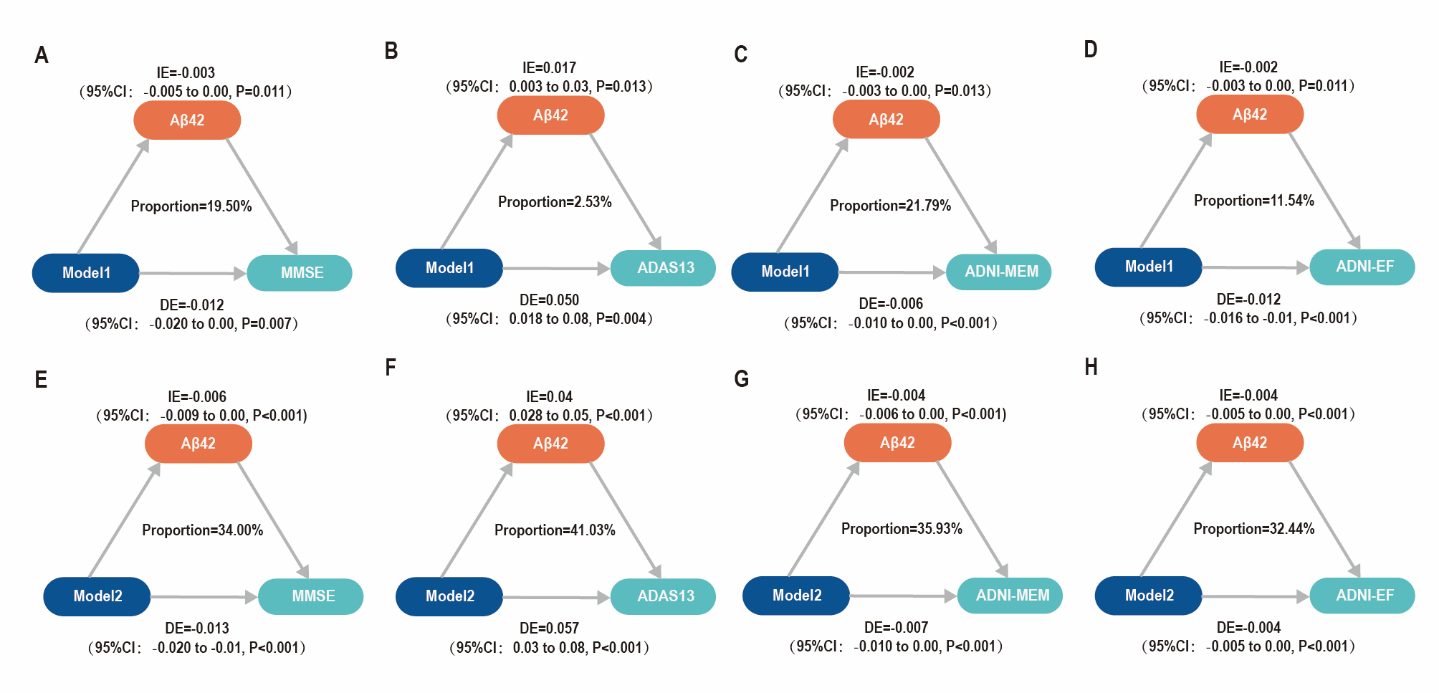


Supplementary Figure 5. CSF Aβ42 moderated the effects of MDRS on MMSE, ADAS and cognitive domains as cognitive outcomes in ADNI study


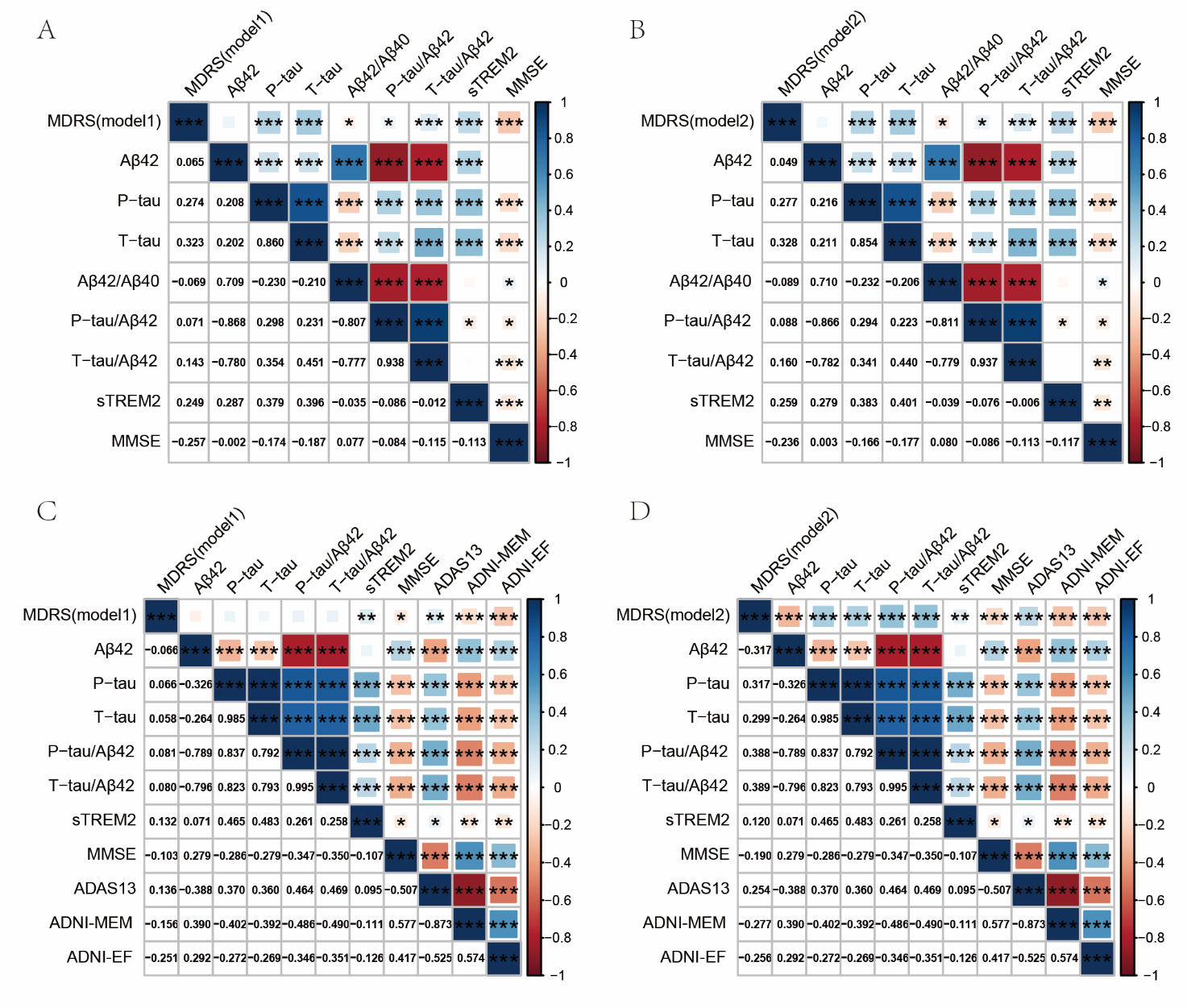


Supplementary Figure 6. Correlations between MDRS, CSF biomarkers and cognition in CABLE and ADNI study


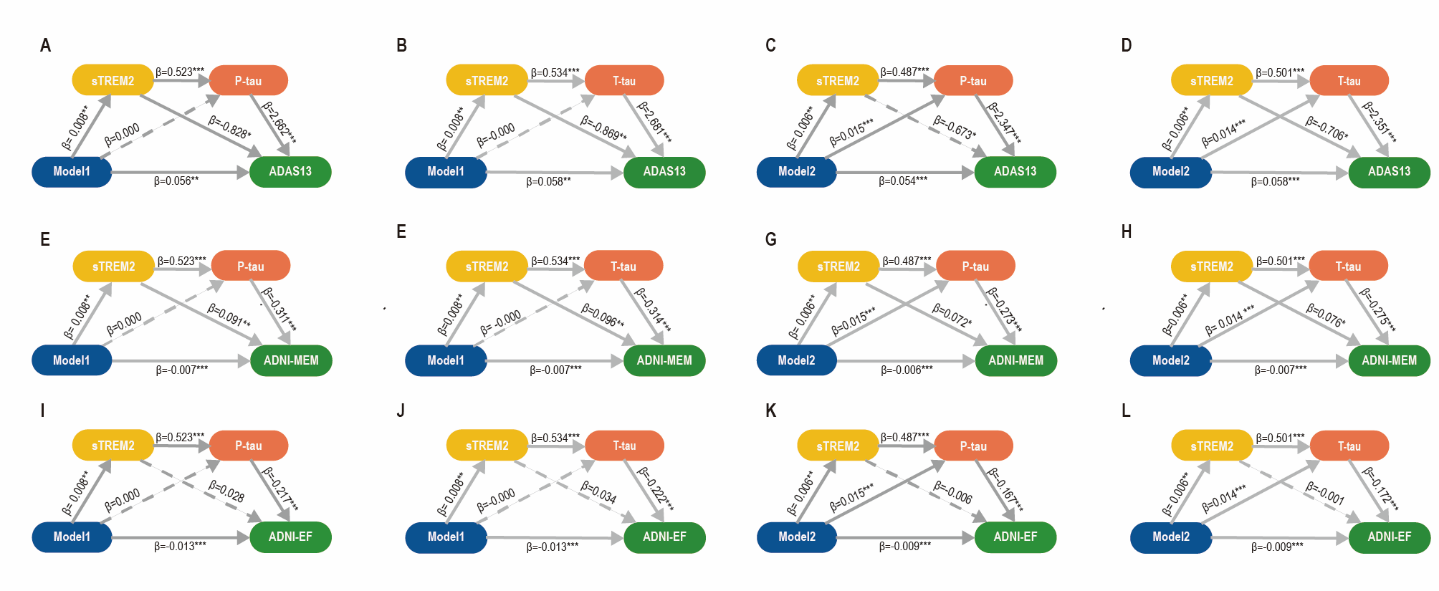


Supplementary Figure 7. CSF sTrem2 and tau-related biomarkers moderated the effects of MDRS on ADAS and cognitive domains as cognitive outcomes in ADNI study


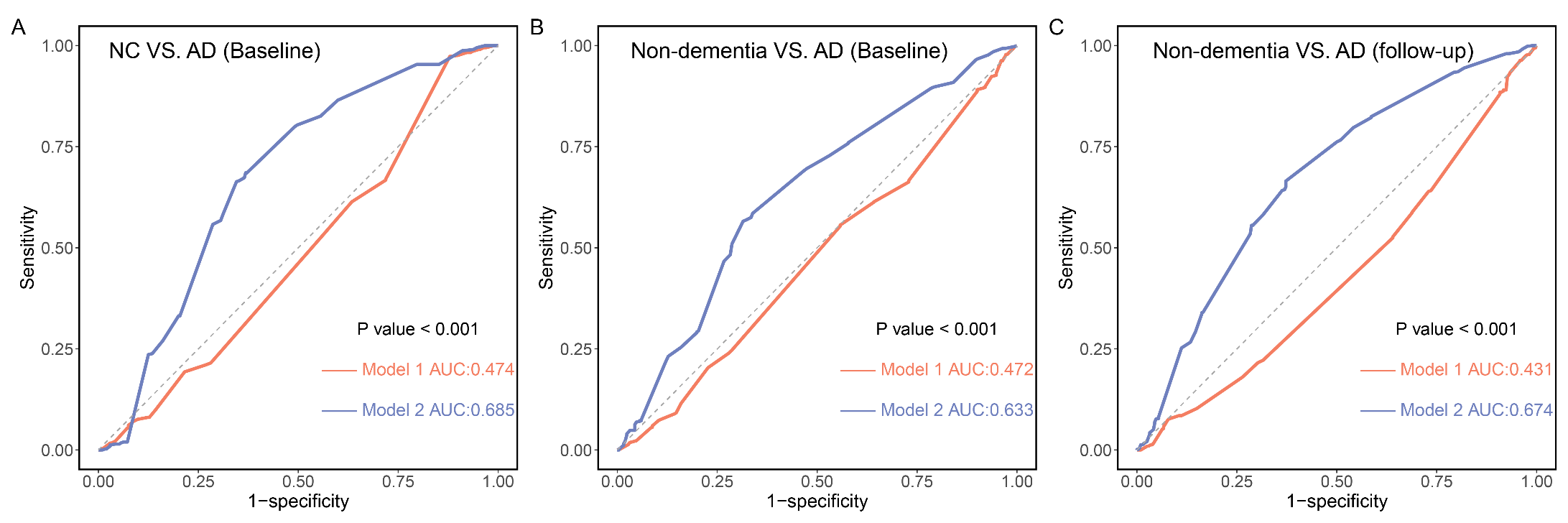


**Supplementary Figure 8. Accuracy of MDRS in distinguishing between AD and NC/non-dementia subjects**
